# Efficacy and Phytotoxicity Assessment of Successive Application of Methyl Bromide and Cold Treatment on Export Strawberry Fruits

**DOI:** 10.3390/insects12110990

**Published:** 2021-11-03

**Authors:** Bong-Su Kim, Ji-Eun Choi, Deuk-Soo Choi, Jeong-Oh Yang

**Affiliations:** Department of Plant Quarantine, Animal and Plant Quarantine Agency (APQA), Gimcheon 39660, Korea; bskim79@korea.kr (B.-S.K.); cje1993@naver.com (J.-E.C.); dschoi@korea.kr (D.-S.C.)

**Keywords:** fumigant, cold treatment, *Drosophila suzukii*, strawberry, combined treatment

## Abstract

**Simple Summary:**

Spotted wing Drosophila, *Drosophila suzukii*, is an important quarantine pest in Korea due to its oviposition behavior. In this study, insecticidal effects of methyl bromide (MB) alone and successive application of methyl bromide and cold treatment were compared to examine their potential to reduce methyl bromide usage and shorten the cold treatment periods. Eggs, larvae, and pupae were subjected to treatment with fumigants to determine the 50 and 99% lethal concentration time values. The results show that, through treatment with methyl bromide plus cold, control can be achieved at lower concentrations than for treatment with methyl bromide alone and with shorter treatment periods than for treatment with cold treatment alone. Phytotoxic effects from successive treatment on strawberry were not observed. Therefore, methyl bromide plus cold treatment can effectively control *D. suzukii* in strawberry without damage to fresh fruits.

**Abstract:**

Recently, spotted wing Drosophila, *Drosophila suzukii*, is globally prevalent and causes agricultural losses to many fruits. To export Korean strawberry, methyl bromide fumigation is required to remove *D. suzukii* infestations, but Korean strawberry farmers are worried about fruit damage because methyl bromide can cause phytotoxicity on fresh commodities. In this report, we assessed the efficacy and phytotoxicity of single and successive application of methyl bromide and cold treatment on an export variety of strawberry to reduce fruit damage. The currently recommended dosage of methyl bromide, 40 g/m^3^ for 3 h at 18 °C, was enough to control all stages of *D. suzukii* without phytotoxicity. A dosage of 20 g/m^3^ of methyl bromide treatment for 3 h, followed by 1 d of cold (0 °C) treatment, showed 100% mortality in all growth stages of *D. suzukii* without fruit damage. Successive application of methyl bromide and cold treatment shows potential as a method of decreasing phytotoxicity and reducing the use of methyl bromide for environmental considerations.

## 1. Introduction

Spotted wing drosophila, *Drosophila suzukii*, is an important quarantine pest in Korea because it can disrupt grape and strawberry exports. Unlike other drosophila species, *D. suzukii* lays eggs inside fruit by damaging the fruit surface [1]. This oviposition behavior makes *D. suzukii* difficult to find through visual inspection by quarantine officers. Therefore, many countries have designated *D. suzukii* a quarantine pest, but *D. suzukii* invasions have been reported recently, including in Europe and America [2,3]. Korean farmers have difficulty exporting fruits such as grape and strawberry, which are potential hosts of *D. suzukii*.

When exported to other countries, Korean strawberries should be fumigated with 40 g/m^3^ of methyl bromide for 3 h at 18 °C. This standard treatment is based on a scientific report by Walse et al. [4]. In that report, all stages including the egg were completely controlled by methyl bromide dosages of more than 34.5 g/m^3^. Similar results were reported by other researchers. Fumigation with 40 g/m^3^ methyl bromide can control other fruit fly species such as the Mediterranean fruit fly, *Ceratitis capitata* [5,6] and the Caribbean fruit fly, *Anastrepha suspense* [7].

Methyl bromide has been used for many decades to control quarantine pests. Because of its short fumigation time, good penetration, and ease of use at low pressure with high efficacy, methyl bromide is an exceptional fumigant for quarantine use [8,9]. Recently, however, methyl bromide has been designated as an ozone-depleting substance, and is now banned in many countries except for quarantine purposes [10]. As a quarantine fumigant, methyl bromide still has a problem. On some fruits and vegetables, methyl bromide can cause damage and loss of quality [11,12]. Korean farmers are also concerned about quality loss of export strawberry after methyl bromide treatment. Several methyl bromide alternative fumigants, such as ethyl formate and phosphine, were developed to reduce environmental hazards and quality loss. However, phosphine and ethyl formate treatment also can cause damage to strawberry. In our previous study, phosphine changed the smell of strawberry, and ethyl formate caused the calyx to wilt [13]. Therefore, more studies should be conducted to develop ethyl formate and phosphine as alternative fumigants to methyl bromide for export strawberry.

Cold treatment is a physical treatment method commonly used on fresh commodities for plant quarantine purposes. The International Plant Protection Convention (IPPC) specified the cold treatment regulation in ISPM 28 as a fruit fly control method. Kim et al. [14] reported the 99.9968% (Probit-9) standard of cold treatment on *D. suzukii*; however, treatment time requires 8 to 10 days to achieve Probit-9 mortality at 1 °C. The quality of some strawberry cultivars can be decreased even at low temperature [15], so reducing treatment time is essential to maintain fruit quality.

In this paper, we compared the insecticidal effect of methyl bromide alone and successive application of methyl bromide and cold treatment to examine the possibility of reducing methyl bromide usage and shortening the cold treatment periods. Additionally, we also investigated the quality loss of an export strawberry, the ‘*Maehyang*’ cultivar, when treated with methyl bromide alone and with successive application of methyl bromide and cold treatment. Soluble solid content, surface color, weight loss and decay rate were measured to assess the phytotoxicity of each treatment [12].

## 2. Materials and Methods

### 2.1. Tested Plant

Export strawberry (*Fragaria x ananassa*) cultivar, ‘*Maehyang*’, was purchased from a farming association (Jinju-si, Korea). The ‘*Maehyang*’ cultivar was harvested when surface color was between 60 to 70 percent reddish. Purchased strawberry was immediately stored at 5 ± 1 °C in a refrigerator (UDScientific, Seoul, Korea) until use to maintain quality [16], and stored strawberries were consumed within a week.

### 2.2. Tested Insect

Spotted wing Drosophila, *Drosophila suzukii*, was used as a test insect in this experiment. *D. suzukii* was reared at Chonnam National University, Korea, in an insect breeding cage (W30 cm × D30 cm × H30 cm, MegaView Science Co., Ltd., Taipei, Taiwan) at 28 ± 1 °C and 60 ± 10% RH with continuous light. An artificial diet made with vinegar was half-filled in plastic insect breeding dish (ø100 × 40 mm, SPL Life science, Pocheon-si, Korea) and used to induce egg-laying and to breed larvae. Sugared water with 20% concentration also was supplied to maintain adults. All insects were transferred to the Animal and Plant Quarantine Agency, Korea, before the experiment, and reared under the same conditions. The egg, larva, and pupa stages, which could be found inside fruit, were used for this experiment.

### 2.3. Fumigant and Fumigation Procedures

Methyl bromide (98.5%) was purchased from Nonghyup Chemicals, Seongnam-si, Korea. Tested insects were contained in insect breeding dish with numbers of more than 30, and three dishes were used in each treatment for statistical analysis. Methyl bromide was injected from cylinder to 10 L Tedlar’s bag (SKC Inc., Eighty Four, PA, USA) and vaporized for small scale fumigation trials. A 125-L gas-tight fumigation chamber (Uni BNC, Goyang-si, Korea) was prepared, and a strawberry fruit box was loaded inside chamber with 16% filling ratio. An electric fan with 100 mm diameter (Uni BNC, Goyang-si, Korea) was placed at the bottom of the chamber to circulate gas. After fruit and insect loading, each chamber was closed and well-sealed with duct tape, and placed inside a reefer container (28 m^3^) to regulate temperature. The temperature of the reefer container was fixed at 18 °C, and fumigant was injected with a gas-tight syringe (2 L, SGE Analytical, Victoria, Australia).

### 2.4. Measurement of Fumigant Concentration

Mixed gas inside the fumigation chamber was collected with 50 mL gas-tight syringe to measure fumigant concentration inside the chamber. Collected gas was injected inside a 1 L volume Tedlar’s bag (SKC Inc., Eighty Four, PA, USA). The sampling periods were 0.5, 1, 2, and 3 h after fumigation. Sampled gas was assessed with gas chromatography (GC 7890A, Agilent Technologies, Palo Alto, CA, USA). Methyl bromide was separated by Rtx-5 column (15 m × 250 μm × 1 μm, Restek, Bellefonte, PA, USA) with split mode (10:1), and concentration was measured by flame ionization detector (FID). The temperature of the injector, oven, and detector were 200 °C, 200 °C, and 250 °C, respectively. The injection volume and flow rate for methyl bromide were 70 μL and 1.5 mL/min, respectively. The methyl bromide concentration was calculated based on peak areas compared with external standard.

### 2.5. Determination of Concentration Time (Ct) Product

The efficacy of fumigant is generally related to both concentration and fumigation time, so fumigant efficacy can be proportioned with a concentration x time (Ct) product [8]. The Ct product of the fumigant was calculated using the equation of [17]:

Ct = ∑(Ci + Ci+1) (ti+1 − ti)/2

where:

C is the fumigant concentration (mg/L)
  t is time of exposure (h)
  i is the order of measurement
  Ct is the concentration × time (mg h/L)

### 2.6. Efficacy and Phytotoxicity Assessment

After fumigation, the chamber was opened and ventilated until the gas concentration was decreased to less than the threshold limit value (TLV). Tested insects were moved to the rearing room, and the mortality of the larvae was checked using a microscope (S8 APO, Leica, Wetzlar, Germany) at 3 days after treatment to avoid knock-down effect. The mortality of eggs and pupae was checked by counting the emerged numbers.

The tested strawberry was transferred to a 5 ± 1 °C refrigerator to maintain quality, and investigated for phytotoxicity after 3, 7, 10, and 14 days. Strawberry firmness was measured with a fruit firmness tester (HPE II FFF, Heinrich Bareiss GmbH, Oberdischingen, Germany) equipped with a 5 mm diameter flat probe (Heinrich Bareiss GmbH, Oberdischingen, Germany). Sample fruit was placed horizontally, and firmness at the middle of the strawberry fruit was measured and recorded. Thirty strawberries were used to measure firmness for each treatment. Soluble solid content was measured with a refractometer (GMK-703AC, G-won Hitech, Seoul, Korea). Strawberry fruit was ground with a pestle, and gauze-filtered juice was dropped on the sensor of a refractometer. Measured soluble solid contents were expressed as °brix [18]. Surface color was measured with a colorimeter (TECHKON SPECTRO DENS, TECHKON GmbH, Konigstein, Germany), and expressed as L, a, and b values.

Weight loss of fruit was evaluated by periodic weighing of each treatment sample. For each test, 30 fruits per treatment were used.

### 2.7. Statistical Analysis

The lethal concentration time 50 (LCt_50_) and 99 (LCt_99_) value of fumigant on *D. suzukii* was determined by Probit analysis [19]. The calculated LCt values for each treatment were compared and expressed as synergistic ratios (SRs) [20].

SR = (LCt of methyl bromide alone)/(LCt of successive treatment)

where:

SR = 1 describes additive action,
  SR < 1 describes antagonism,
  SR > 1 describes synergism.

One-way analysis of variance (ANOVA) using the SPSS program (IBM Corporation 2016) was performed to check the differences in phytotoxic effect from each fumigation method.

## 3. Results

### 3.1. Comparative Efficacy of Methyl Bromide Alone and Methyl Bromide plus Cold Treatment on D. suzukii

The LCt_50_ value of a single treatment of methyl bromide on *D. suzukii* was highest at the larva stage (17.18 mg h/L) and lowest at the egg stage (6.37 mg h/L), whereas the LCt_99_ value of a single treatment of methyl bromide on *D. suzukii* was highest at the egg stage (85.41 mg h/L) and lowest at the pupa stage (32.04 mg h/L) (Table 1).

The cold treatment (0 °C) on *D. suzukii* showed more than 62% mortality at all stages when stored for 1 day. Complete mortality was achieved in the egg and larva stages when stored for more than 5 days, and 3 days were required to completely control the pupa stage (Table 2).

The LCt_50_ value for successive treatment with methyl bromide and cold treatment (0 °C, 1 day) on *D. suzukii* was highest at the pupa stage (20.03 mg h/L) and lowest at the larva stage (6.37 mg h/L). The LCt_99_ value for *D. suzukii* was highest at the egg stage (85.41 mg h/L) and lowest at the pupa stage (32.04 mg h/L), as were the LCt_50_ values. (Table 3). The synergistic ratio of methyl bromide and cold treatment differed by growth stage, from 1.77 to 11.30, but all stages showed enhanced efficacy compared to that from a single treatment of methyl bromide (Table 4).

### 3.2. Efficacy and Phytotoxicity Assessment of Methyl Bromide and Successive Treatment on Strawberry

Methyl bromide showed 100% efficacy on larva, pupa, and the adult stage of *D. suzukii*, but not on egg stage when samples were treated with 20 mg/L for 3 h. The successive treatment of methyl bromide and cold treatment induced 100% mortality in all stages of *D. suzukii* (Table 5). No phytotoxic effects were observed from either a single treatment or successive treatment (Table 6 and Table 7).

## 4. Discussion

Recently, as methyl bromide has been restricted for environment protection purposes, sustained efforts to develop new methyl bromide alternative techniques have been made [21,22]. Ethyl formate and phosphine, which are being developed as representative chemical fumigant alternatives to methyl bromide, already have been commercialized in Korea but are not widely supplied due to the irreplaceable properties of methyl bromide, its low price, and short fumigation period [23]. In physical control, techniques such as hypoxic treatment, irradiation, and controlled atmosphere temperature treatment system (CATTS) are being studied as methyl bromide alternatives, but still more studies are required [24,25]. There also have been ongoing studies on the combination of physical and chemical treatment methods. Haritos et al. tried to enhance the efficacy of ethyl formate by using a carbon dioxide (CO_2_) combination treatment [26]. Kim et al. reported the synergistic effect of oxygen on phosphine and ethyl formate to control *Phthorimaea operculella* [27]. Thermal treatment can be a good option to increase the efficacy of a fumigant [28]. Underwood et al. reported the enhancement of fumigant efficacy at increased temperature [29]. However, this attempt requires a more careful approach because, in general, while fumigant efficacy is enhanced at increased temperature [30], the quality of fruits or vegetables is maintained under appropriate, normally low-temperature conditions [31]. This study was implemented as an extension of these physicochemical successive treatment studies.

In this study, a single treatment of methyl bromide showed no damage on strawberry; therefore, it is assumed that using a current fumigation standard (40 mg/L methyl bromide for 3 h at 18 °C) will not cause fruit damage to the export strawberry cultivar. The result was consistent with Walse et al. [4], which reported that methyl bromide does not cause any phytotoxicity to strawberries and there were no fruit quality changes in several strawberry cultivars. Though current treatment causes no damage on strawberry, the development of treatment alternatives to methyl bromide is still important because of environmental issues. The successive treatment of methyl bromide and cold treatment showed increased efficacy against *D. suzukii* without phytotoxic effects on Korean strawberry cultivars. The synergistic ratio of the pupa stage was lower than those of the other stages, but in harvested strawberry, the pupa stage is more likely to be found because of body color and the fact that 82~93% of pupae are found in soil [32]; hence, it has been determined that the quarantine risk from pupa can be lower than the risk at other stages. The synergistic ratios of egg and larva stages are greatly increased, so these stages can be controlled with less methyl bromide usage. Benschoter also reported that efficacy was enhanced with successive treatments of methyl bromide and cold treatment [7].

Successive treatment of fumigant and cold treatment can be applied not only for methyl bromide but also for other fumigants. Though single treatment was not enough to control *D. suzukii* without damage, ethyl formate and phosphine with physical treatment can decrease phytotoxicity. Lee et al. confirmed that the reduced phytotoxicity in orange with successive treatment of ethyl formate and cold treatment [20], and Zhang et al. reported that successive treatment with phosphine and cold treatment can reduce phytotoxicity in white chrysanthemums [33]. According to Liu, there was no phytotoxicity in strawberry with 500 or 1000 ppm of phosphine in low-temperature conditions [34]. Kwon et al. recently reported the synergism of cold treatment plus ethyl formate on *D. suzukii*; that combination treatment of ethyl formate and cold treatment showed significantly increased efficacy compared to cold treatment alone [35]. Using physical treatment as a complementary method can be a good option to overcome the limits of chemical phytosanitary treatment.

Although the efficacy of methyl bromide on *D. suzukii* was increased when it was followed by cold treatment, we are not certain that this enhancement originated from the synergistic effects of the two treatments because the cold treatment itself showed good efficacy on *D. suzukii*. Additionally, synergism itself can differ depending on the targeted insect species. Regarding the synergistic effect of concurrent treatments of ethyl formate and phosphine, Lee et al. and Yang et al. reported positive synergistic effects on *Aphis gossypii* Glover and *Planococcus citri* Risso [20,23]. However, Lee et al. reported no synergism between ethyl formate and phosphine to control *Tetranychus urticae* Koch [36]. To utilize this successive treatment as a method of reducing methyl bromide use, further studies on the mode of action against several insect pests will be required.

## 5. Conclusions

Methyl bromide did not induce phytotoxic effects on the Korean strawberry cultivar, ‘*Maehyang*’, when applied at with currently used dosage, 40 g/m^3^ for 3 h at 18 °C, and completely controlled all growth stages of *D. suzukii*. A lower dosage of methyl bromide was required to achieve similar efficacy on *D. suzukii* compared to a single treatment without fruit damage when methyl bromide treatment was followed by cold treatment. Successive treatment with methyl bromide and cold treatment can be a good alternative fumigation method to reduce methyl bromide usage for environmental considerations.

## Figures and Tables

**Table 1:** Lethal concentration time for *D. suzukii* exposed to methyl bromide fumigant.

Stages	*n*	LCt_50_ (mg h/L) (95% CL ^a^)	LCt_99_ (mg h/L) (95% CL)	Slope ± SE ^b^	*df*	χ^2^
Egg	4425	6.37 (3.34–9.46)	85.41 (60.11–148.47)	2.06 ± 0.24	8	946.72
Larva	4453	17.18 (6.87–29.24)	39.44 (24.52–352.26)	3.55 ± 1.08	6	2217.20
Pupa	4177	8.83 (2.32–13.49)	32.04 (19.27–416.69)	4.15 ± 1.09	6	1576.57

^a^ CL = confidence limit. ^b^ SE = standard error.

**Table 2:** Mortality of *D. suzukii* exposed to cold treatment (0 °C) for 0, 1, 2, 3, 5 d in cold chamber.

Treatment Method	Egg		Larva		Pupa	
	*n*	Mortality ± SE ^a^ (%)	*n*	Mortality ± SE (%)	*n*	Mortality ± SE (%)
Cold trt ^b^ 0 day	607	5.5 ± 0.5 a	653	5.7 ± 0.3 a	600	4.5 ± 1.3 a
Cold trt 1 day	1756	62.1 ± 7.6 b	1075	82.9 ± 5.0 b	699	69.2 ± 2.9 b
Cold trt 2 day	1395	85.6 ± 2.7 c	1152	97.4 ± 0.6 c	1119	96.7 ±0.9 c
Cold trt 3 day	1776	96.9 ± 0.5 d	1206	99.3 ± 0.3 c	1142	100.0 ± 0.0 c
Cold trt 5 day	1983	100.0 ± 0.0 d	1863	100.0 ± 0.0 c	1668	100.0 ± 0.0 c

^a^ SE = standard error; ^b^ trt = treatment; Mean values within each column followed by the same letter are not significantly different (*p* < 0.05).

**Table 3:** Lethal concentration time for *D. suzukii* exposed to successive application of methyl bromide and cold treatment for 1 d.

Stages	*n*	LCt_50_ (mg h/L) (95% CL ^a^)	LCt_99_ (mg h/L) (95% CL)	Slope ± SE ^b^	*df*	χ^2^
Egg	5051	2.94 (0.92–5.08)	7.87 (4.30–10.62)	3.00 ± 0.40	6	150.18
Larva	4241	1.83 (0.01–4.13)	3.49 (0.15–6.05)	4.57 ± 1.16	6	15.68
Pupa	5898	3.09 (0.49–5.63)	18.06 (12.88–30.48)	3.03 ± 0.63	7	777.48

^a^ CL = confidence limit. ^b^ SE = standard error.

**Table 4:** Synergistic ratios of methyl bromide and cold treatment on each growth stage of *D. suzukii*.

Stages	Synergistic Ratio ^a^ LCt_50_	Synergistic Ratio ^b^ LCt_99_
Egg	2.17	10.85
Larva	9.39	11.30
Pupa	2.86	1.77

^a^ Synergistic ratio (SRs) = LCt_50_ of methyl bromide alone/LCt_50_ of successive treatment; ^b^ Synergistic ratio (SRs) = LCt_99_ of methyl bromide alone/LCt_99_ of successive treatment.

**Table 5:** Mortality of *D. suzukii* exposed to methyl bromide for 3 h at 18 °C in 12 L desiccator.

Fumigant Concn. (mg/L)	Ct Product (mg h/L)	Egg		Larva		Pupa		Adult	
		*n*	Mortality ± SE ^a^ (%)	*n*	Mortality ± SE (%)	*n*	Mortality ± SE (%)	*n*	Mortality ± SE (%)
Control	-	486	6.6 ± 1.3 a	476	3.6 ± 1.5 a	575	4.6 ± 1.1 a	43	0.0 ± 0.0 a
40.0	87.3	792	100.0 ± 0.0 c	605	100.0 ± 0.0 b	687	100.0 ± 0.0 b	51	100.0 ± 0.0 b
20.0	41.7	614	87.8 ± 5.4 b	590	100.0 ± 0.0 b	681	100.0 ± 0.0 b	47	100.0 ± 0.0 b
20.0 + Cold trt 1 d	41.7	758	100.0 ± 0.0 c	598	100.0 ± 0.0 b	559	100.0 ± 0.0 b	49	100.0 ± 0.0 b

^a^ SE = standard error; Mean values within each column followed by the same letter are not significantly different (*p* < 0.05).

**Table 6:** Phytotoxicity on strawberry (‘*Maehyang*’ cultivar) exposed to 40 mg/L methyl bromide for 3 h (Ct product = 87.3 mg h/L) at 18° C in 500 L container, then stored at 5 ± 1 °C.

Storage Period (Days)	Treatment	Deterioration	Mean Surface Color			Weight Reduction Ratio (%)	Sugar Content (%brix)
			L	a	b		
3	Control	0.0 ± 0.0 a	38.9 ± 1.5 a	152.6 ± 2.4 a	152.1 ± 5.8 a	0.0	8.5 ± 0.6 a
	40 mg/L	0.0 ± 0.0 a	41.4 ± 1.2 a	143.9 ± 6.9 a	146.2 ± 1.8 a	0.0	9.0 ± 0.4 a
7	Control	0.0 ± 0.0 a	37.4 ± 2.0 a	153.8 ± 8.1 a	150.3 ± 6.1 a	0.9 ± 0.3 a	8.0 ± 0.3 a
	40 mg/L	0.0 ± 0.0 a	39.4 ± 3.3 a	151.5 ± 5.0 a	150.6 ± 9.3 a	1.0 ± 0.1 a	8.8 ± 0.6 a
10	Control	0.0 ± 0.0 a	40.6 ± 0.4 a	165.5 ± 6.5 a	162.4 ± 6.1 a	0.6 ± 0.1 a	8.1 ± 0.1 a
	40 mg/L	0.0 ± 0.0 a	42.9 ± 2.4 a	164.1 ± 7.8 a	162.1 ± 8.8 a	0.5 ± 0.0 a	9.0 ± 0.4 a
14	Control	0.0 ± 0.0 a	38.5 ± 1.0 a	160.9 ± 5.5 a	157.1 ± 5.7 a	1.0 ± 0.1 a	7.1 ± 0.3 a
	40 mg/L	0.0 ± 0.0 a	40.9 ± 2.0 a	158.0 ± 7.8 a	155.6 ± 9.8 a	1.2 ± 0.2 a	8.1 ± 0.4 a

Mean values within each column followed by the same letter are not significantly different (*p* < 0.05).

**Table 7:** Phytotoxicity on strawberry (‘*Maehyang*’ cultivar) exposed to 20 mg/L methyl bromide for 3 h (Ct product = 41.7 mg h/L) at 18 °C and cold treatment for 1 day at 0 °C in 500 L container, then stored at 5 ± 1 °C.

Storage Period (Days)	Treatment	Deterioration	Mean Surface Color			Weight Reduction Ratio (%)	Sugar Content (%brix)
			L	a	b		
3	Control	0.0 ± 0.0 a	45.3 ± 3.3 a	146.0 ± 6.8 a	139.5 ± 1.6 a	0.0	8.1 ± 0.1 a
	20 mg/L + Cold trt 1 day	0.0 ± 0.0 a	44.2 ± 1.6 a	139.9 ± 4.8 a	143.7 ± 5.1 a	0.0	8.5 ± 0.2 a
7	Control	0.0 ± 0.0 a	39.0 ± 1.8 a	139.6 ± 3.4 a	137.8 ± 5.1 a	1.8 ± 1.0 a	7.6 ± 0.3 a
	20 mg/L + Cold trt 1 day	0.0 ± 0.0 a	41.8 ± 3.4 a	144.9 ± 2.5 a	144.1 ± 4.0 a	1.3 ± 0.6 a	7.5 ± 0.6 a
10	Control	0.0 ± 0.0 a	40.7 ± 2.2 a	146.1 ± 2.8 a	144.8 ± 2.1 a	1.5 ± 0.5 a	7.7 ± 0.2 a
	20 mg/L + Cold trt 1 day	0.0 ± 0.0 a	39.6 ± 2.6 a	147.2 ± 5.3 a	146.1 ± 6.2 a	1.0 ± 0.2 a	8.4 ± 0.5 a
14	Control	0.0 ± 0.0 a	38.6 ± 1.8 a	141.5 ± 7.8 a	139.8 ± 7.6 a	0.8 ± 0.1 a	7.4 ± 0.1 a
	20 mg/L + Cold trt 1 day	0.0 ± 0.0 a	37.0 ± 0.9 a	141.0 ± 6.7 a	139.0 ± 7.7 a	2.2 ± 1.9 a	7.1 ± 0.6 a

Mean values within each column followed by the same letter are not significantly different (*p* < 0.05).

## Data Availability

There is no supplementary information to reveal, all the information is contained in this manuscript.
